# Optimisation of DNA extraction from the crustacean *Daphnia*

**DOI:** 10.7717/peerj.2004

**Published:** 2016-05-10

**Authors:** Camila Gonçalves Athanasio, James K. Chipman, Mark R. Viant, Leda Mirbahai

**Affiliations:** School of Biosciences, University of Birmingham, Birmingham, United Kingdom

**Keywords:** Genomics, Omics, Epigenetics, High throughput sequencing

## Abstract

*Daphnia* are key model organisms for mechanistic studies of phenotypic plasticity, adaptation and microevolution, which have led to an increasing demand for genomics resources. A key step in any genomics analysis, such as high-throughput sequencing, is the availability of sufficient and high quality DNA. Although commercial kits exist to extract genomic DNA from several species, preparation of high quality DNA from *Daphnia* spp. and other chitinous species can be challenging. Here, we optimise methods for tissue homogenisation, DNA extraction and quantification customised for different downstream analyses (e.g., LC-MS/MS, Hiseq, mate pair sequencing or Nanopore). We demonstrate that if *Daphnia magna* are homogenised as whole animals (including the carapace), absorbance-based DNA quantification methods significantly over-estimate the amount of DNA, resulting in using insufficient starting material for experiments, such as preparation of sequencing libraries. This is attributed to the high refractive index of chitin in *Daphnia’s* carapace at 260 nm. Therefore, unless the carapace is removed by overnight proteinase digestion, the extracted DNA should be quantified with fluorescence-based methods. However, overnight proteinase digestion will result in partial fragmentation of DNA therefore the prepared DNA is not suitable for downstream methods that require high molecular weight DNA, such as PacBio, mate pair sequencing and Nanopore. In conclusion, we found that the MasterPure DNA purification kit, coupled with grinding of frozen tissue, is the best method for extraction of high molecular weight DNA as long as the extracted DNA is quantified with fluorescence-based methods. This method generated high yield and high molecular weight DNA (3.10 ± 0.63 ng/µg dry mass, fragments >60 kb), free of organic contaminants (phenol, chloroform) and is suitable for large number of downstream analyses.

## Introduction

*Daphnia spp.* are considered keystone species in both lakes and ponds and are well-studied in terms of their ecology and response to stressors, both under laboratory conditions and in the field (Lampert, 2011). For example, *Daphnia magna* has been used extensively for ecotoxicological assays for many years (OECD, 2004; OECD, 2012) while *Daphnia pulex* has been listed as a model system for biomedical research by the National Institutes of Health, USA (Colbourne et al., 2011). Furthermore, both species have been proposed as model organisms for environmental genomics, toxicogenomics and epigenetics studies (Eads, Andrews & Colbourne, 2008; Colbourne et al., 2011; Harris, Bartlett & Lloyd, 2012; Miner et al., 2012). *Daphnia* are key model organisms used for research into the molecular mechanisms of phenotypic plasticity, adaptation and microevolution (Giessler, Mader & Schwenk, 1999; Van Doorslaer et al., 2009; Messiaen et al., 2010; Messiaen et al., 2013; Geerts et al., 2015). The extensive use of *Daphnia* spp. in a wide range of research fields has motivated the development and optimisation of several omics technologies to probe the molecular machinery within these species (Taylor et al., 2008; Dircksen et al., 2011; Colbourne et al., 2011). The resulting growth of genomic resources for *Daphnia* spp., coupled with the dramatic reduction in costs and accessibility of sequencing technologies and other genomic tools, has fuelled their increasing use in environmental genomics, toxicogenomics and evolutionary biology (Pfrender, Spitze & Lehman, 2000; Omilian & Lynch, 2009; Orsini, Spanier & DE Meester, 2012; Hochmuth et al., 2015).

Isolation of high quality genomic DNA from biological material is a critical first step in determining the success of downstream genomics and epigenomics studies. Despite the development and use of several standard protocols for reproducibly extracting high quality, high yield DNA from a range of biological materials in a high throughput manner (Kan et al., 2004; Yu et al., 2008), we and others have experienced numerous challenges applying these procedures to the chitinous crustacean, *Daphnia* spp., often requiring a large number of individuals per biological replicate (Vandegehuchte et al., 2009; Menzel et al., 2011; Brakovska & Škute, 2013; Routtu et al., 2014). Conventional DNA extraction methodology can be unreliable when applied to *D. magna* and related species, potentially due to the chitinous exoskeleton termed the carapace. Thus, there remains an urgent need to develop a DNA extraction technique for *Daphnia* spp. that is compatible with different downstream analyses. Ideally, techniques for DNA isolation should have high extraction efficiency, yielding high length DNA that is free of contamination.

Considering this need, the aim of this study was to identify suitable methods of genomic DNA extraction as well as selecting optimal methods for quantification of the extracted DNA. During this study we compared several methods for tissue disruption as well as for extracting the DNA from the homogenised tissue. In addition, we compared the gold standard method for DNA quantification, based on using DNA-specific fluorescent dyes, with convenient UV absorbance methods. Finally, we explained the unreliability of absorbance-based methods for quantification of *D. magna* DNA.

## Materials and Methods

### *Daphnia* culture and sample collection

*Daphnia magna* Straus, 1820 Bham 2 clones were used to optimise the DNA extraction methods. However, the methods used here could also be applied to other microcrustacean*s*, as tested in our laboratory (data not shown). *D. magna* were maintained in a 16:8 h light:dark photoperiod and temperature of 20 ± 1 °C, in high hardness COMBO medium (HH COMBO). Media were prepared using a protocol adapted from Baer & Goulden (1998) and Kilham et al. (1998), and renewed once a week. Animals were fed every other day with *Chlorella vulgaris* at a concentration of ≈27,550 cells of algae per individual *Daphnia*. For each sample, a single 28 day-old *D. magna* was used. Each *Daphnia* was dissected rapidly to remove any embryos present in the brood chamber, since the number of embryos in the brood chamber can vary, resulting in high variation in the amount of starting tissue. Immediately following dissection, the animals were preserved for subsequent DNA extraction using one of two methods, either placing in RNA*later* (Ambion) and incubation overnight at 4 °C and stored at −80 °C or flash freezing in liquid nitrogen and stored at −80 °C. The overnight 4 °C incubation allowed the RNA*later* to penetrate and preserve the tissue.

### Homogenisation methods

Three methods of homogenisation and lysis were evaluated: (i) a 2010 Geno/Grinder bead-based tissue homogeniser (SPEX SamplePrep, Stanmore, UK; 1.4 mm ceramic beads, 1,750 strokes/minute for 40 s); homogenisation was conducted in the presence of the respective lysis solution for each of the four extraction protocols described below. (ii) Homogenisation using a plastic pellet pestle (Sigma Aldrich, Dorset, UK); immediately after removing from liquid nitrogen, samples were ground to a fine powder, lysis solution was added, and the extraction protocols were followed according to the methods listed below. (iii) Overnight proteinase K digestion; lysis buffer was added to each sample and incubated overnight (16 h). The last method left the carapace undigested at the bottom of the tube and hence care was taken to avoid its disturbance and the carryover of any fragment to the lysate.

**Table 1:** DNA yield measured by fluorescence-based method of quantification (SYBR Green I) represented by the mean ± SEM for ng of DNA per µg of *Daphnia* dry mass. Missing values are due to salt precipitation from RNA *later* solution. Comparisons were made between the groups with same homogenisation method and with same extraction method. Same letters indicate the groups that differ statistically.

Preservation	Extraction method	Homogenisation method		
		Ceramic bead homogenisation	Plastic pellet pestles	Proteinase K digestion
Liquid Nitrogen	Agencourt DNAdvance	5.40 ± 0.12^a^^,^^b^^,^^d^	4.10 ± 0.23^c^	2.77 ± 0.18^d^
	CTAB method	3.13 ± 0.71^b^	3.52 ± 0.79^c^	2.29 ± 0.17
	MasterPure DNA	5.45 ± 0.91^a^^,^^b^^,^^e^	3.10 ± 0.63^e^	2.93 ± 0.42^e^
	ZR genomic DNA	1.65 ± 0.10^a^	1.26 ± 0.09^c^	0.97 ± 0.05
RNA*later* solution	Agencourt DNAdvance	4.07 ± 0.20	4.37 ± 0.08^f^	3.93 ± 0.20^g^
	CTAB method	2.26 ± 0.03	4.13 ± 0.87^f^	–
	MasterPure DNA	–	–	–
	ZR genomic DNA	2.07 ± 0.16	1.52 ± 0.16^f^	0.97 ± 0.03^g^

**Notes.**

*P*-values are indicated accordantly.

a *p* < 0.0001.

b *p* < 0.05.

c *p* < 0.05.

d *p* < 0.01.

e *p* < 0.05.

f *p* < 0.01.

g *p* < 0.01.

### Extraction methods

Four methods of DNA extraction were evaluated (*n* = 3 biological replicate/extraction method), as presented in Table 1. Three of the methods were commercially available kits, (I) Agencourt DNAdvance (Beckman Coulter, Indianopolis, IN, USA) employing magnetic beads, (II) MasterPure DNA purification (Epicentre, Madison, WI, USA), using protein precipitation followed by isopropanol DNA precipitation and (III) ZR Genomic DNA™-Tissue MicroPrep (Zymo Research, Irvine, CA, USA), a column-based method (IV) while the fourth was a modified version of the CTAB extraction method (Doyle & Doyle, 1987). For the commercial kits, the manufacturer’s instructions were followed with some modifications, as described below. Extracted DNA samples were stored in −80 °C.

*I. Agencourt DNAdvance:* DNA extraction was performed following the manufacturer’s instructions. Lysis solution (200 µl) (Agencourt DNAdvance kit) containing proteinase K (7 µl at 40 mg/ml) was added to each sample. For the bead-based and pestle homogenisation methods, samples were incubated at 55 °C for 15 min, mixing briefly every 5 min. For the proteinase K digestion method, samples were incubated overnight at 55 °C. Following the homogenisation step, DNA was extracted using magnetic beads and the protocol provided by the manufacturer.

*II. MasterPure DNA purification kit:* DNA was extracted using a MasterPure DNA purification kit (Epicentre, Madison, WI, USA). The protocol was modified from the manufacturer’s instructions in that ‘Tissue and Cell Lysis solution’ (300 µl) and proteinase K (1 µl at 50 µg/µl) were added to each sample. For the bead-based and pestle homogenisation methods, the samples were incubated at 65 °C for 15 min, mixing briefly every 5 min. For the proteinase K digestion method, samples were incubated overnight at 65 °C. After incubation, samples were cooled to 37 °C and RNase cocktail (3 µl, RNase $A=500\hspace*{1em}\mathrm{U}/\mathrm{ml}$; RNase T1 = 20,000 U/ml; Ambion) was added to each sample. Following 30 min of incubation at 37 °C, samples were placed on ice for 5 min. The protein precipitation and DNA purification was achieved according to the manufacturer’s protocol.

*III. ZR Genomic DNA™-Tissue MicroPrep*: DNA was extracted following the manufacturer’s protocol for DNA extraction from solid tissues. Homogenisation was performed as previously described using 200 µl of digestion buffer containing a final concentration of 20 mg/ml of proteinase K. For the bead-based and pestle homogenisation methods, samples were incubated at 55 °C for 1 h. For the proteinase K digestion method, samples were digested overnight at the same temperature. Genomic lysis buffer (700 µl, ZR Genomic DNA™-Tissue MicroPrep) was added to each digested sample which was then transferred to the Zymo-Spin™ IC Column and the DNA was extracted according to the manufacturer’s protocol.

*IV. CTAB extraction method*: DNA was extracted using a modified CTAB protocol (Doyle & Doyle, 1987). For the bead-based and pestle homogenisation methods, 300 µl CTAB buffer (2% hexadecyltrimethyl ammonium bromide CTAB, 1.4 M NaCl, 20 mM EDTA, 100 mM Tris-HCl pH 8, 0.2% *β*-mercaptoethanol, 50 µg of proteinase K) was added to each sample. The solutions were incubated at 65 °C for 1 h, mixing briefly every 20 min. For the proteinase K digestion method, samples were incubated overnight in 300 µl of CTAB buffer at 65 °C. Following the incubation step, DNA was extracted from all the samples using 500 µl of chloroform: isoamyl alcohol (24:1) (Sigma Aldrich, Dorset, UK). The samples were centrifuged and the top aqueous layer containing the nucleic acids was transferred to a clean 1.5 ml microcentrifuge tube.

To ensure complete removal of RNA contamination from the DNA samples, we performed a RNA digestion step using RNase cocktail (Ambion, Paisley, UK). This method combines the RNA degradation activities of RNase A and RNase T1. The RNase A specifically hydrolyses RNA at C and U residues while RNase T1 specifically hydrolyses RNA at G residues. The combine use of RNase A and RNase T1 results in higher level of RNA degradation than using either of the enzymes alone without interfering with DNA purification, quantification and any downstream analyses. All samples were incubated with 3 µl of RNase cocktail for 30 min at 37 °C. Next, a further DNA extraction was performed using chloroform:isoamyl alcohol (24:1). In a clean microcentrifuge tube, 1 volume of isopropanol was added to the transferred aqueous layer to precipitate the DNA; each sample was incubated for at least 1 h at −80 °C. Next, each sample was washed twice, once with 100% ice-cold ethanol (1 ml) and once with 70% ice-cold ethanol. The DNA pellet was air-dried and resuspended in 30 µl of Tris-EDTA buffer, and stored at −80 °C.

This study comprises a full factorial design containing two sample preservation methods, three tissue homogenisation/lysis methods, and four DNA extraction methods.

### Assessment of DNA quantity and quality

The extracted DNA was quantified with: (1) 8000 UV-Vis spectrophotometer (NanoDrop, Wilmington, DE, USA) and (2) SYBR Green DNA I dye (Thermo Fisher Scientific, Paisley, UK) using an Infinite^®^ 200 PRO microplate reader (Tecan, Männedorf, Switzerland). These represent an absorbance- and a fluorescence-based method of DNA quantification, respectively. DNA yield was reported as ng of DNA per µg of dry mass of single *Daphnia*, where the dry mass of a single 28-day old *Daphnia* was measured as 295.5 ± 15.9 µg (mean ± SEM). The quality and integrity of DNA samples and potential RNA contamination were also assessed using a 1% agarose gel in TBE buffer containing Midori Green Advance DNA Stain (Nippon Genetics, Dueren, Germany). Same amount of DNA for each sample was loaded onto the gel and electrophoresis was performed at 80 V for 50 min. The quality of the extracted DNA, for the two methods of extraction that resulted in the highest yield of DNA, was further investigated with 2200 TapeStation (Agilent Technologies, Stockport, Cheshire, UK) and genomic DNA ScreenTape. To compare the DNA integrity, the average DNA fragment sizes were classified as low (150 to 4,000 bp) or high (4,000 to >60,000 bp) molecular weight DNA. Results are expressed as percentage of fragments for each category.

### Investigation of carapace

Chitin can cause interference with the 260 nm reads due to its refraction index, causing the overestimation of the amount of DNA (Azofeifa, Arguedas & Vargas, 2012). To assess interference of carapace debris with DNA quantification, moulted carapaces were collected from the bottom of culture vessels and were treated with antibiotics (50 mg/l tetracycline, streptomycin and ampicillin) to remove bacteria, as a large microbiome has been associated with *Daphnia* (Qi et al., 2009). The treated carapaces were homogenised using ceramic beads and were processed according to the same methodology described in MasterPure DNA purification section. As the overestimation of DNA seems to be related to the method of homogenisation using grinding and not the methods of extraction, only MasterPure DNA purification was applied to these samples to test this hypothesis. The processed carapace were analysed with both methods, absorbance and fluorescence-based, of DNA quantification.

### Statistical analyses

The software IBM SPSS Statistics 22 was used to assess the normal distribution of the data. From 40 different groups (method of preservation vs. method of homogenisation vs. method of extraction vs. method of quantitation) only 3 did not presented normal distribution applying the Shapiro–Wilk test. Therefore, the subsequent analyses were performed assuming a Gaussian distribution although with caution due to the small sample size. Two-way ANOVA with Sidak’s multiple comparison test was applied for the comparison between the concentration values obtained with fluorescence and absorbance analysis. One-way ANOVA with Tukey’s multiple comparison test was used to compare the yield of the different groups. These analyses were performed using GraphPad Prism 6 software.

## Results

In total this study evaluated 20 combinations of treatment steps, a full factorial design comprising of two sample preservation methods, three tissue homogenisation/lysis methods, and four DNA extraction methods. Furthermore, DNA samples were analysed for quality and quantity using agarose-based methods and fluorescence/absorbance-based methods, respectively (Table 1).

### DNA quantity

**Figure 1:**
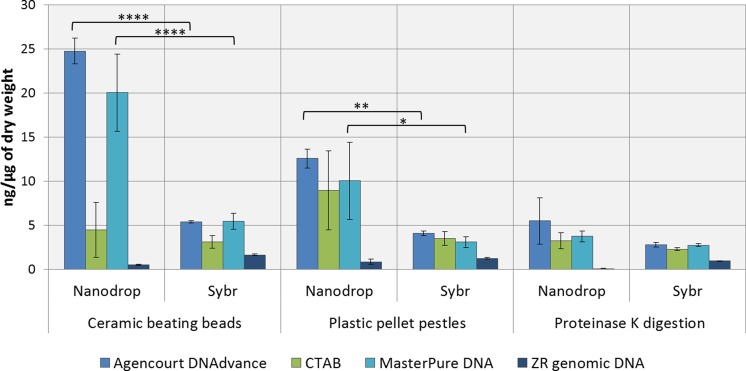
Average DNA concentration measured with NanoDrop 8000 and SYBR Green I. Error bars indicate standard error of the mean. ^∗^*p* < 0.05; ^∗∗^*p* < 0.01; ^∗∗∗∗^*p* < 0.0001; No statistically significant differences were observed for CTAB probably due to greater data variability.

DNA concentrations were overestimated when using the convenient absorbance-based method of DNA quantification (i.e., NanoDrop spectrophotometry) for the samples homogenised with ceramic beating beads and plastic pellet pestles, except for DNA extracted with the ZR genomic DNA kit (Fig. 1). The apparent DNA concentrations were usually 3–4 fold higher than when measured with the gold standard fluorescence-based method of SYBR Green I (Two-way ANOVA, *p* < 0.0001). Measurements made with NanoDrop also presented greater variability, expressed as the standard error of the mean in Fig. 1. The difference in the measured concentration of DNA between the absorbance and fluorescence-based methods of quantifications may be attributed to the presence of chitin from the *Daphnia*’s carapace. For the ZR genomic DNA kit, a column based method, measurements with NanoDrop underestimated the concentration of DNA.

DNA concentrations are expressed as ng of DNA per µg of dry mass (Table 1) as measured with SYBR Green DNA I dye. As demonstrated in Table 1, the method of preservation, RNA*later* versus liquid nitrogen, did not significantly (One-way ANOVA, *p* > 0.05) affect DNA yield. However, all methods of extractions that included a precipitation step (e.g., 1 volume of isopropanol in CTAB and MasterPure DNA extraction methods) also resulted in co-precipitation of salts from RNA*later* solution which can interfere with downstream analysis. Both Agencourt DNAdvance and ZR genomic DNA only use the ethanol step to wash the DNA trapped either on the magnetic beads or on the column, therefore, the salts are not precipitated with the DNA.

Tissue disruption and homogenisation is the first step in DNA extraction. In this study we compared three methods of tissue homogenisation: ceramic beating beads, plastic pellet pestles and overnight digestion with proteinase K. Ceramic beating beads resulted in the maximum disruption and release of DNA, followed closely by samples ground using plastic pestles and finally proteinase K digestion (Table 1; For samples extracted using MasterPure: ceramic beating beads > proteinase K, *p* < 0.01).

Regarding the DNA extraction methods, CTAB, Agencourt DNAdvance and MasterPure DNA Purification Kit resulted in similar yields when homogenised with plastic pellet pestles. Agencourt DNAdvance and MasterPure DNA purification kit performed better for the homogenisation with ceramic beating beads. The column based extraction ZR Genomic DNA micro kit, consistently yielded the least DNA of the four methods tested regardless of the homogenisation procedure (Table 1).

**Figure 2:**
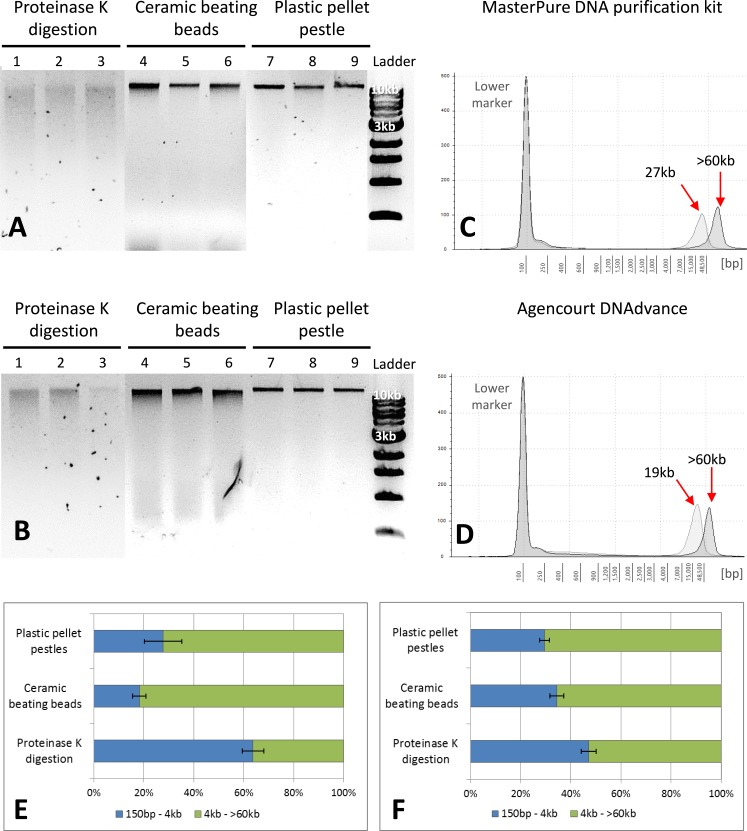
Quality assessment of DNA samples extracted with MasterPure DNA purification kit and Agencourt DNAdvance. (A) 1% agarose gel for MasterPure samples. (B) 1% agarose gel for DNAdvance samples; same amount of DNA was loaded onto each lane. Lanes 1–3 present fragmented DNA spread along the lane, while lanes 4–9 present majority of the DNA in a distinct band above the 10 kb marker position. (C) TapeStation results for MasterPure. (D) TapeStation results for DNAdvance. Dark grey—Homogenisation with plastic pestle; Light grey—homogenisation ceramic beating beads. (E) DNA fragments distribution for MasterPure samples. (F) DNA fragments distribution for DNAdvance samples. Error bars in (E) and (F) indicate standard error of the mean.

### DNA purity and quality

DNA purity is usually estimated using the ratios between the absorbance values for A260/A280 and A260/A230 wavelengths measured with a spectrophotometer. However, for *Daphnia*, due to high non-specific absorbance at A260, these ratios cannot be used to assess the purity of DNA. Nevertheless, the magnitude of differences between the DNA concentrations estimated by spectrophotometry and the fluorescence-based method of SYBR Green I can detect contaminants.

Gel electrophoresis was used to assess DNA integrity and RNA contamination (Figs. 2A and 2B). As shown in Figs. 2A and 2B, no RNA contamination was detectable after RNA digestion with RNase cocktail regardless of the methods of tissue preservation, homogenisation and extraction. However, the method of homogenisation did affect the integrity of DNA. All the samples submitted to overnight proteinase K digestion showed high fragmentation in comparison to the other two methods of homogenisation. Ceramic beating beads resulted in slightly higher fragmentation of DNA than plastic pestle ground samples. Based on gel electrophoresis, DNA fragmentation was caused by the homogenisation method and was unaffected by the extraction method.

The three methods, CTAB, MasterPure DNA purification and Agencourt DNAdvance resulted in the highest yields of DNA, irrespective of the method used for homogenising the tissue. CTAB is a time consuming protocol that often employs the use of phenol and chloroform for the extraction step and can easily contaminate the DNA sample. Thus, quality of the extracted DNA was further assessed for MasterPure DNA purification and Agencourt DNAdvance only (two methods × three methods of tissue homogenisation) using Genomic DNA ScreenTape and the 2200 Tapestation (Fig. 2). Samples were analysed for both percentage of low and high molecular weight fragments and for the average size of high molecular weight DNA fragments (Figs. 2C–2F). Fragmentation percentage analysis indicated that overnight proteinase K digestion resulted in around 50% of the fragments below 4 kb for both extraction methods, with average fragment size of 25 kb for the high molecular weight DNA. This indicates that tissue digestion with proteinase K caused severe degradation of DNA, making it unsuitable for downstream techniques that require longer DNA fragments such as Pacbio and mate pair sequencing. However, it has to be noted that this method only digests the tissue, leaving the carapace intact. Thus, as the carapace can be removed prior to DNA extraction, it is the only method of tissue disruption where DNA quantification based on absorbance is very similar to the gold standard fluorescence method of DNA quantification, SYBR Green I (Two-way ANOVA, *p* > 0.05).

The DNA extracted with ceramic beating beads had 65% and 81% of the fragments classified as high molecular weight, with the average fragment size of 27 kb and 19 kb for the MasterPure kit and Agencourt DNAdvance, respectively. For plastic pellet pestle homogenisation, 70% of the DNA fragments were classified as high molecular weight for both isolation methods with the average size greater than 60 kb.

To confirm the interference of the carapace with absorbance measurements, the same DNA extraction procedure was performed (MasterPure kit) with isolated carapaces. Samples were then quantified with NanoDrop and SYBR Green I. No DNA was observed with the fluorescence dye. However according to the NanoDrop measurements the samples apparently yielded on average 748.6 ± 22.1 (mean ± SEM) ng of DNA.

## Discussion

### Liquid nitrogen versus *RNAlater*: the effect of tissue preservation method on the quality and quantity of extracted DNA

We assessed the quality of the DNA extracted from tissues preserved in RNA*later* and snap-frozen in liquid nitrogen. RNA*later* is a storage solution with a high concentration of salts. It is used to stabilize and protect the RNA from degradation and minimizes the need to immediately process or freeze the samples. Therefore, it is the preferred method of sample preservation for field studies (Gorokhova, 2005). Our results indicate that both methods can result in extraction of high quality DNA from *Daphnia* (Table 1). However, we demonstrated that DNA extraction methods with an alcohol-based precipitation step are not ideal for extracting DNA from tissues that have been preserved in RNA*later* as the alcohol will result in co-precipitation of DNA with the salts present in RNA*later*. Many enzymes used in downstream applications can be inhibited by excessive concentrations of salts (Wilson, 1997). Therefore, for samples that are preserved in RNA *later*, it is recommended to use alternative methods of extraction, such as bead based methods of DNA extraction.

### Tissue homogenisation and disruption impacts the quality and yield of DNA

Three methods of tissue homogenisation and disruption were assessed: (i) overnight digestion with proteinase K, (ii) ceramic beating beads and (iii) plastic pellet pestle. Each method has advantages and disadvantages, influencing their suitability for downstream analysis (Table 2).

**Table 2:** Summary of the advantageous and disadvantageous associated with each method of tissue homogenisation.

Method of tissue homogenisation	Advantages	Disadvantages	Applicability
**Ceramic bead homogenisation**	High DNA yield, rapid tissue homogenisation, compatibility with RNA and metabolite sample preparations	Overestimation of DNA concentration using absorbance based methods of quantification (e.g., NanoDrop) due to carapace interference, requires quantification with fluorescence dye, mechanical DNA fragmentation	Suitable for high throughput sample homogenisation and automation, simultaneous extraction of RNA, DNA and metabolites, suitable for downstream analyses requiring average fragment size (e.g., RRBS, WGBS, Next-Seq and HiSeq)
**Plastic pellet pestles**	High quality and high DNA yield, large fragments of DNA	Overestimation of DNA concentration using absorbance based methods of quantification (e.g., NanoDrop) due to carapace interference, time consuming, possible cross-contamination	Suitable for downstream analyses requiring large DNA fragments (long-read sequencing)
**Proteinase K digestion**	Low level of carapace contamination, absorbance based methods of quantitation are less affected	Highly fragmented DNA, low DNA yield, time consuming	Suitable for PCR based methods

**Notes.**

Abbreviations WGBS Whole genome bisulfite sequencing RRBS Reduced representation bisulfite sequencing

The ceramic beating beads yielded higher amounts of DNA compared to other homogenisation methods, except when coupled with CTAB extraction. Although expensive, this method is rapid, high throughput and reduces the risk of cross contamination (Leite, Magan & Medina, 2012). Although time-consuming, plastic pellet pestles are also an effective and inexpensive method of tissue homogenisation. As demonstrated in Fig. 2, both methods resulted in generation of high quality, high yield and high molecular weight DNA (yield based on DNA extraction with MasterPure: ceramic beads (5.45 ± 0.91 ng/ug dry mass) > plastic pellet pestle (3.10 ± 0.63) > proteinase K (2.93 ± 0.42)).

**Figure 3:**
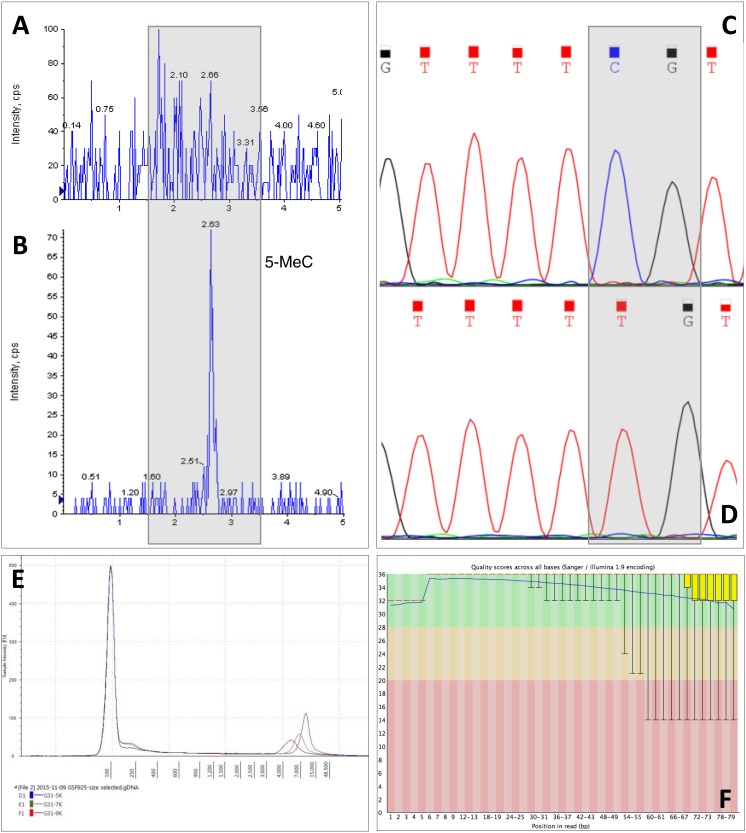
Example of downstream analyses of DNA samples. (A) Chromatogram of hydrolysed *Daphnia* DNA sample extracted with CTAB. (B) Chromatogram of hydrolysed *Daphnia* DNA sample extracted with MasterPure DNA purification kit. Shadowed area indicates 5-methylcytosine position. (C) Artificially methylated DNA generated from DNA extracted from *Daphnia* with MasterPure DNA purification kit. Sample was methylated using SssI methyltransferase (NEB, USA), treated with sodium bisulfite to preserve DNA methylation patterns, amplified using PCR and sequenced on the ABI 3730. (D) Unmethylated DNA generated from *Daphnia* with whole genome amplification (Sigma Aldrich, Dorset, UK) using MasterPure DNA purification kit. Shadowed area indicates cytosine-guanine position. (E) DNA fragment size selection (3 kb–9 kb) for mate pair library construction. (F) Quality scores for mate pair sequencing data.

However, both methods introduced a contaminant causing an increase in the 260 nm absorbance and over-estimation of DNA concentration by 4–5 fold. We have demonstrated that this interference is caused by the *Daphnia’s* carapace. *Daphnia’s* body is enclosed by a double walled shell, largely made of the polysaccharide chitin (Ebert, 2005). Chitin has a high refractive index around 260 nm and shows an anomalous dispersion for wavelengths lower than 300 nm (Azofeifa, Arguedas & Vargas, 2012). Therefore, the chitin structure and its optical properties can explain the anomalous DNA quantification. We have not observed any detrimental effects on downstream techniques, such as high throughput DNA sequencing, due to the presence of chitin in the DNA samples (Fig. 3). However, if *Daphnia* has been homogenised as whole (carapace included) for DNA extraction, DNA must be quantified with fluorescence-based methods (SYBR Green I) rather than absorbance-based methods.

This claim was further evidenced by using *Daphnia* tissue digested overnight with proteinase K, the carapace remaining intact while the tissue was completely digested. After removal of the carapace and extraction of the DNA, there was no statistically significant difference between the concentrations of DNA measured with absorbance-based methods or fluorescence-based methods (DNA concentrations extracted with MasterPure; absorbance = 3.74 ± 0.61 ng/µg, fluorescence = 2.76 ± 0.18 ng/µg, Two-way ANOVA, *p* > 0.05). However, the downside of using overnight proteinase K digestion is that the method is time consuming, results in lower yield of DNA compared to the other two methods of homogenisation and also it results in fragmentation of DNA (Fig. 2). DNA integrity is extremely important for long read sequencing technologies, such as Oxford Nanopore, mate pair sequencing and Pacbio (Wang et al., 2015). Mate pair sequencing is a method for long-insert libraries applied specially for *de novo* assembly and structural variation detection (Illumina, 2014). Mate pair libraries were successfully constructed using *Daphnia* DNA samples homogenised with plastic pellet pestles and extracted with MasterPure kit containing inserts ranging from 3 kb to 9 kb. High quality was evidenced since the mean Q scores for every sample we sequenced was above 33 (Fig. 3).

**Figure 4:**
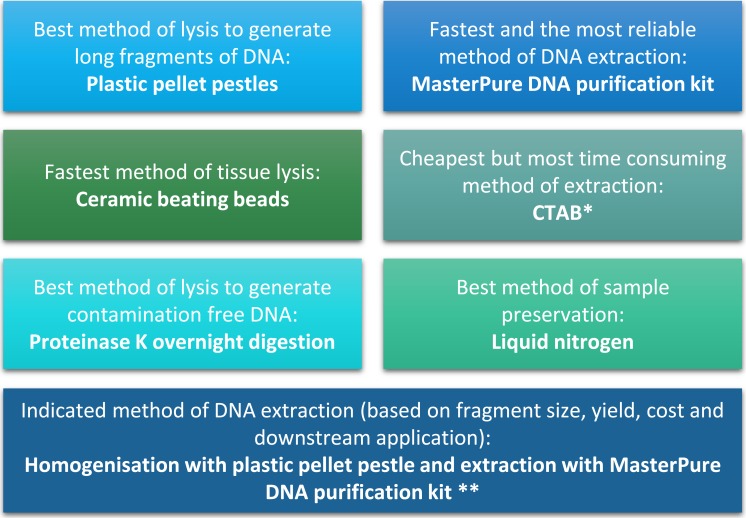
Summary of conclusion regarding the best methods of tissue homogenisation and DNA extraction. ^∗^ CTAB is not suitable for samples that will be used for mass spectrometry. ^∗∗^ Samples need to be quantified with fluorescence-based method.

### DNA extraction method and impact on downstream analyses

The ZR genomic DNA purification columns resulted in the lowest yields of DNA, but removed carapace contamination (Table 1 and Fig. 1). DNA concentrations were underestimated by absorbance in comparison with fluorescence.

CTAB isolation protocol has been suggested for *Daphnia* samples and can remove polysaccharide contamination, at least for plant samples (Doyle & Doyle, 1987; Fang, Hammar & Grumet, 1992; Michiels et al., 2003). However, for *Daphnia* samples CTAB did not remove carapace contamination, despite the high DNA concentration. Also, DNA extracted with CTAB cannot be analysed by mass spectrometry for base modifications, an important technique for epigenetic studies, since it interferes with the ionisation process causing a high background signal (Figs. 3A and 3B). Furthermore, CTAB is a time consuming protocol that can introduce organic contamination, especially through the chloroform extraction step, and can cause loss of DNA during the ethanol washing steps. It also employs the use of hazardous chemicals; however it is an inexpensive technique.

The MasterPure DNA kit and Agencourt DNAdvance resulted in similar yields and similarly retained carapace components. Agencourt DNAdvance is expensive, but is more suited for robotic preparation systems and is compatible with *RNAlater* preserved samples. Due to the lower cost of MasterPure method of extraction, we recommend this method as an ideal method of DNA extraction from *Daphnia*. As demonstrated in our laboratory, DNA extracted with this method is suitable for use in a range of downstream analyses, such as whole genome amplification, PCR, mass spectrometry, mate pair sequencing, Nextseq and HiSeq analyses, as long as DNA is quantified with fluorescent based methods (Fig. 3).

### Remarks and conclusions

Difficulties with DNA extraction for *Daphnia* have been previously reported (Brakovska & Škute, 2013), such as the need for high number of *Daphnia* to obtain sufficient amount of DNA (Vandegehuchte et al., 2009; Menzel et al., 2011; Routtu et al., 2014). This can limit the scope of experiments by increasing the number of animals required. In terms of DNA yield, we obtained 284 µg to 2,061 µg per adult *D. magna*, depending on homogenisation and isolation method. We showed that it is possible to obtain sufficient high quality DNA for most molecular techniques from single *Daphnia* using MasterPure method of DNA extraction coupled to blue pellet pestle method of tissue homogenisation. This make large-scale studies with several end-points feasible.

The quality and quantity of extracted DNA are crucial factors in determining the success of downstream analyses. For *Daphnia* we have shown that tissue homogenisation methods, DNA extraction methods and DNA quantification methods can significantly impact the quality and quantity of the extracted DNA and need to be considered during the experimental design (Fig. 4). However, based on our results it appears that the homogenisation step has a higher impact on the quality of the DNA compared to the method of DNA extraction. Both ceramic beating beads and plastic pellet pestles generated high yield and high molecular weight DNA. However, plastic pellet pestles (average fragment size: >60 kb) are cheaper and the average fragment size is larger than the ceramic beating beads (average fragment size: 23 kb). Finally, we have created a flowchart (Fig. 4) to help researchers decide on the best method of tissue homogenisation and extraction from chitinous crustacea based on their downstream method of analysis.

## Additional Information and Declarations

### Data Availability

The following information was supplied regarding data availability: Raw data is deposited in Figshare:
https://figshare.com/s/4ecbc0e5be441ffc1fbf.

## References

[ref-1] Azofeifa DE, Arguedas HJ, Vargas WE (2012). Optical properties of chitin and chitosan biopolymers with application to structural color analysis. Optical Materials.

[ref-2] Baer KN, Goulden CE (1998). Evaluation of a high-hardness COMBO medium and frozen algae for *Daphnia magna*. Ecotoxicology and Environmental Safety.

[ref-3] Brakovska A, Škute N (2013). Optimisation of DNA extraction and RAPD-PCR amplification for population genetic analysis of *Daphnia cucullata* Sars, 1862 (Crustacea: Cladocera). Acta Biologica Universitatis Daugavpiliensis.

[ref-4] Colbourne JK, Pfrender ME, Gilbert D, Thomas WK, Tucker A, Oakley TH, Tokishita S, Aerts A, Arnold GJ, Basu MK, Bauer DJ, Cáceres CE, Carmel L, Casola C, Choi JH, Detter JC, Dong Q, Dusheyko S, Eads BD, Fröhlich T, Geiler-Samerotte KA, Gerlach D, Hatcher P, Jogdeo S, Krijgsveld J, Kriventseva EV, Kültz D, Laforsch C, Lindquist E, Lopez J, Manak JR, Muller J, Pangilinan J, Patwardhan RP, Pitluck S, Pritham EJ, Rechtsteiner A, Rho M, Rogozin IB, Sakarya O, Salamov A, Schaack S, Shapiro H, Shiga Y, Skalitzky C, Smith Z, Souvorov A, Sung W, Tang Z, Tsuchiya D, Tu H, Vos H, Wang M, Wolf YI, Yamagata H, Yamada T, Ye Y, Shaw JR, Andrews J, Crease TJ, Tang H, Lucas SM, Robertson HM, Bork P, Koonin EV, Zdobnov EM, Grigoriev IV, Lynch M, Boore JL (2011). The ecoresponsive genome of *Daphnia pulex*. Science.

[ref-5] Dircksen H, Neupert S, Predel R, Verleyen P, Huybrechts J, Strauss J, Hauser F, Stafflinger E, Schneider M, Pauwels K, Schoofs L, Grimmelikhuijzen CJP (2011). Genomics, transcriptomics, and peptidomics of *Daphnia pulex* neuropeptides and protein hormones. Journal of Proteome Research.

[ref-6] Doyle J, Doyle J (1987). A rapid DNA isolation procedure for small quantities of fresh leaf tissue. Phytochemical Bulletin.

[ref-7] Eads BD, Andrews J, Colbourne JK (2008). Ecological genomics in *Daphnia*: stress responses and environmental sex determination. Heredity.

[ref-8] Ebert D (2005). Introduction to *Daphnia* Biology. Ecology, epidemiology, and evolution of parasitism in daphnia.

[ref-9] Fang G, Hammar S, Grumet R (1992). A quick and inexpensive method for removing polysaccharides from plant genomic DNA. BioTechniques.

[ref-10] Geerts AN, Vanoverbeke J, Vanschoenwinkel B, Van Doorslaer W, Feuchtmayr H, Atkinson D, Moss B, Davidson TA, Sayer CD, De Meester L (2015). Rapid evolution of thermal tolerance in the water flea *Daphnia*. Nature Climate Change.

[ref-11] Giessler S, Mader E, Schwenk K (1999). Morphological evolution and genetic differentiation in *Daphnia* species complexes. Journal of Evolutionary Biology.

[ref-12] Gorokhova E (2005). Effects of preservation and storage of microcrustaceans in RNA*later* on RNA and DNA degradation. Limnology and Oceanography: Methods.

[ref-13] Harris KDM, Bartlett NJ, Lloyd VK (2012). *Daphnia* as an emerging epigenetic model organism. Genetics Research International.

[ref-14] Hochmuth JD, De Meester L, Pereira CMS, Janssen CR, De Schamphelaere KAC (2015). Rapid adaptation of a *Daphnia magna* population to metal stress is associated with heterozygote excess. Environmental Science & Technology.

[ref-15] Illumina (2014). Nextera mate pair library preparation kit. http://www.illumina.com/content/dam/illumina-marketing/documents/products/datasheets/datasheet_nextera_mate_pair.pdf.

[ref-16] Kan C-W, Fredlake CP, Doherty EAS, Barron AE (2004). DNA sequencing and genotyping in miniaturized electrophoresis systems. Electrophoresis.

[ref-17] Kilham SS, Kreeger DA, Lynn SG, Goulden CE, Herrera L (1998). COMBO: a defined freshwater culture medium for algae and zooplankton. Hydrobiologia.

[ref-18] Lampert W (2011). Daphnia: development of a model organism in ecology and evolution.

[ref-19] Leite GM, Magan N, Medina Á (2012). Comparison of different bead-beating RNA extraction strategies: an optimized method for filamentous fungi. Journal of Microbiological Methods.

[ref-20] Menzel S, Bouchnak R, Menzel R, Steinberg CEW (2011). Dissolved humic substances initiate DNA-methylation in cladocerans. Aquatic Toxicology.

[ref-21] Messiaen M, De Schamphelaere KAC, Muyssen BTA, Janssen CR (2010). The micro-evolutionary potential of *Daphnia magna* population exposed to temperature and cadmium stress. Ecotoxicology and Environmental Safety.

[ref-22] Messiaen M, Janssen CR, De Meester L, De Schamphelaere KAC (2013). The initial tolerance to sub-lethal Cd exposure is the same among ten naïve pond populations of *Daphnia magna*, but their micro-evolutionary potential to develop resistance is very different. Aquatic Toxicology.

[ref-23] Michiels A, Van den Ende W, Tucker M, Van Riet L, Van Laere A (2003). Extraction of high-quality genomic DNA from latex-containing plants. Analytical Biochemistry.

[ref-24] Miner BE, De Meester L, Pfrender ME, Lampert W, Hairston NG (2012). Linking genes to communities and ecosystems: *Daphnia* as an ecogenomic model. Proceedings of the Royal Society B: Biological Sciences.

[ref-25] Omilian AR, Lynch M (2009). Patterns of intraspecific DNA variation in the *Daphnia* nuclear genome. Genetics.

[ref-26] Organisation for Economic Co-operation and Development (2004). OECD guidelines for the testing of chemicals, Section 2, Test No. 202: *Daphnia* sp. Acute immobilisation test.

[ref-27] Organisation for Economic Co-operation and Development (2012). OECD guidelines for the testing of chemicals, Section 2, Test No. 211: *Daphnia magna* reproduction test.

[ref-28] Orsini L, Spanier KI, DE Meester L (2012). Genomic signature of natural and anthropogenic stress in wild populations of the waterflea *Daphnia magna*: validation in space, time and experimental evolution. Molecular Ecology.

[ref-29] Pfrender ME, Spitze K, Lehman N (2000). Multi-locus genetic evidence for rapid ecologically based speciation in *Daphnia*. Molecular Ecology.

[ref-30] Qi W, Nong G, Preston JF, Ben-Ami F, Ebert D (2009). Comparative metagenomics of *Daphnia* symbionts. BMC Genomics.

[ref-31] Routtu J, Hall MD, Albere B, Beisel C, Bergeron RD, Chaturvedi A, Choi J-H, Colbourne J, De Meester L, Stephens MT, Stelzer C-P, Solorzano E, Thomas WK, Pfrender ME, Ebert D (2014). An SNP-based second-generation genetic map of *Daphnia magna* and its application to QTL analysis of phenotypic traits. BMC Genomics.

[ref-32] Taylor NS, Weber RJM, Southam AD, Payne TG, Hrydziuszko O, Arvanitis TN, Viant MR (2008). A new approach to toxicity testing in *Daphnia magna*: application of high throughput FT-ICR mass spectrometry metabolomics. Metabolomics.

[ref-33] Vandegehuchte MB, Kyndt T, Vanholme B, Haegeman A, Gheysen G, Janssen CR (2009). Occurrence of DNA methylation in *Daphnia magna* and influence of multigeneration Cd exposure. Environment International.

[ref-34] Van Doorslaer W, Stoks R, Duvivier C, Bednarska A, De Meester L (2009). Population dynamics determine genetic adaptation to temperature in *Daphnia*. Evolution.

[ref-35] Wang M, Beck CR, English AC, Meng Q, Buhay C, Han Y, Doddapaneni HV, Yu F, Boerwinkle E, Lupski JR, Muzny DM, Gibbs RA (2015). PacBio-LITS: a large-insert targeted sequencing method for characterization of human disease-associated chromosomal structural variations. BMC Genomics.

[ref-36] Wilson IG (1997). Inhibition and facilitation of nucleic acid amplification. Applied and Environmental Microbiology.

[ref-37] Yu S, Geng J, Zhou P, Wang J, Chen X, Hu J (2008). New hydroxyapatite monolithic column for DNA extraction and its application in the purification of *Bacillus subtilis* crude lysate. Journal of Chromatography. A.

